# Recent Advances towards the Clinical Application of Stem Cells for Retinal Regeneration

**DOI:** 10.3390/cells1040851

**Published:** 2012-10-18

**Authors:** Silke Becker, Hari Jayaram, G. Astrid Limb

**Affiliations:** 1 Ocular Biology and Therapeutics, Institute of Ophthalmology, University College London, 11-43 Bath Street, London EC1V 9EL, UK; Email: h.jayaram@ucl.ac.uk (H.J.); g.limb@ucl.ac.uk (G.A.L.); 2 NIHR Biomedical Research Centre at Moorfields Eye Hospital NHS Foundation Trust and UCL Institute of Ophthalmology, London EC1V 2PD, UK

**Keywords:** stem cells, retina, retinal pigment epithelium, photoreceptors, retinal ganglion cells, retinal degenerative diseases, transplantation, translational research

## Abstract

Retinal degenerative diseases constitute a major cause of irreversible blindness in the world. Stem cell-based therapies offer hope for these patients at risk of or suffering from blindness due to the deterioration of the neural retina. Various sources of stem cells are currently being investigated, ranging from human embryonic stem cells to adult-derived induced pluripotent stem cells as well as human Müller stem cells, with the first clinical trials to investigate the safety and tolerability of human embryonic stem cell-derived retinal pigment epithelium cells having recently commenced. This review aims to summarize the latest advances in the development of stem cell strategies for the replacement of retinal neurons and their supportive cells, the retinal pigment epithelium (RPE) affected by retinal degenerative conditions. Particular emphasis will be given to the advances in stem cell transplantation and the challenges associated with their translation into clinical practice.

## 1. Introduction

Retinal degenerative diseases, which lead to irreversible damage of retinal neurons, represent a major cause of blindness. The most common of these conditions are age-related macular degeneration (AMD), glaucoma and diabetic retinopathy, which together account for more than 25% of all cases of blindness worldwide [1]. Other diseases such as retinitis pigmentosa (RP) can cause debilitating progressive visual impairment at a young age, for which no cure is currently available [2]. Although a large number of patients are at risk of losing vision as a result of these conditions, therapeutic options remain inadequate and retinal degenerative diseases frequently lead to blindness [3]. Whilst neuroprotective strategies may be beneficial for the treatment in the early stages of disease, stem cell transplantation could offer additional therapeutic strategies for patients in advanced stages, in which retinal neurons have been irreparably damaged [3].

Conversely, stem cell therapies for the treatment of retinal degenerations are not yet available and are currently being investigated in preclinical or early clinical trials. These studies aim at assessing the possibility to prevent, or delay the onset of, or even to regenerate and repair established retinal damage through the transplantation of stem cell-derived neurons or retinal pigment epithelium (RPE). With these exciting recent developments in retinal stem cell transplantation, this review aims to summarize the recent advances in stem cell transplantation for the treatment of retinal degenerative diseases.

## 2. Sources of Stem Cells for Transplantation

Although recent studies have demonstrated that resident stem cells can contribute to the regeneration and repair of the central nervous system (CNS) [4], at present there is no evidence for this process within the neural human retina, making damage to the retina irreversible in reality. Due to their pluri- or multipotency and with their progeny capable to differentiate into various different neural cell types, stem cells may serve as a valuable therapeutic tool for the treatment of retinal degenerative conditions in the future. In addition, stem cells have the capacity to self-renew, giving them the potential to generate sufficient numbers of cells for transplantation [5]. Current research focuses on the identification and differentiation of suitable stem cells *in vitro*, their transplantation and replacement of functional retinal neurons and their supportive RPE cells. Various types of stem cells have been studied for use in retinal transplantation therapies, most prominently human embryonic stem cells (hESC), induced pluripotent stem (iPS) cells and adult stem cells such as human Müller stem cells (hMSC).

### 2.1. Human Embryonic Stem Cells

hESC, which are obtained from the inner cell mass of the blastocyst, are capable of unlimited self-renewal while at the same time retaining the ability to differentiate into all the cell types within the human body [6]. Due to these characteristics, it has been suggested that hESC may constitute suitable candidates for the development of retinal cell replacement therapies. Whilst hESC have been shown to differentiate into retinal neurons [7] as well as RPE [8], differentiation protocols required for the generation of specific retinal neuronal cell types are often complex. Although it has been suggested that hESC may differentiate into a neuronal phenotype by default [9], the generation of a specific retinal neuronal phenotype typically requires lengthy and complex protocols [7,10,11].

A major disadvantage concerning the use of hESC for clinical application is the risk of tumorigenesis with reports of teratoma formation following transplantation [12]. Furthermore, hESC frequently display chromosomal abnormalities, which pose further potential risk and will require additional safety studies before clinical application of hESC-based therapies can realistically commence [13]. Since the generation of hESC lines results in the destruction of human embryos, which derive either from embryo donations or surplus tissue from fertility treatments, the use of these cells often raises ethical objections. 

### 2.2. Induced Pluripotent Stem Cells

More recently, iPS cell lines have been generated from adult dermal fibroblasts through retroviral transfection and upregulation of Oct3/4, Klf4, Sox2 and c-Myc [14]. These pluripotent cells share many features with and have been reported to be indistinguishable from embryonic stem cells (ESC) [15]. A major potential advantage of this cell type is that transplantation may be autologous in nature, removing the need for modulation of the host immune system following grafting. Classic methods for the generation of iPS cells require the augmentation of c-Myc, the use of which however is problematic in clinical applications due to its tumorigenic potential. However alternative protocols have been recently reported, which either replace c-Myc with L-Myc [16] or which eliminate the need for the induction of this oncogene [17]. Latest advances in the production of iPS cells have also allowed them to be generated without the use of viral vectors [18].

Despite these recent improvements, the safety of iPS cells needs to be established in future studies before human trials can commence. In particular the risks of inducing potentially tumorigenic pluripotency genes as well as the grafting of cells, which may continue to proliferate or may not be adequately differentiated for transplantation purposes, must be addressed [19]. Although iPS cells share many of the disadvantages and risks of hESC for transplantation, fewer ethical reservations need to be considered, since their generation does not require the use and destruction of embryonic tissue.

### 2.3. Adult Stem Cells

Since the use of hESC and iPS cells for clinical application of retinal cell replacement therapies have several limitations, alternative cell sources, in particular adult stem cells, have been investigated. Mesenchymal stem cells, which can be derived from bone marrow biopsies or isolated from umbilical cord blood and which are able to form bone, cartilage, bone and adipose tissue during embryonic development [20], have been reported to trans-differentiate towards neurons and glial cells [21]. However these findings are not universally accepted and have been disputed in the literature [21]. Whilst mesenchymal stem cells have been studied as a potential source of retinal neurons, their role will most likely be restricted to cell-based neuroprotective strategies rather than for neuronal replacement.

Although the CNS has traditionally been thought to be unable to regenerate and repair tissue damage, this view has recently been challenged and radial glia, which represent the resident neural stem cells in the CNS, have been reported to give rise to neurons in the adult brain [22]. Müller glia, which constitute the radial glia of the retina [23], arise from the same retinal progenitors as retinal neurons [24] and have been reported to retain a population of cells with stem cell characteristics [25]. It has been shown that Müller stem cells can regenerate and repair retinal damage in lower vertebrates by de-differentiation, proliferation and subsequent differentiation into retinal neurons [23].

While the presence of Müller stem cells has been reported in retinae of birds and small mammals [26,27], their regenerative potential appears to be diminished in higher species. Recently Müller glia with stem cell characteristics have also been identified in the adult human retina [25], from which they have be isolated and cultured [28]. These cells display progenitor cell characteristics, including unlimited cell renewal, expression of stem cell and neural precursor markers as well as the potential to differentiate into various retinal neurons [29]. However in contrast to lower vertebrates they appear to lack regenerative potential *in vivo*. While it has been suggested that this may be due to inhibitory factors present in the human retina [30], these remain to be identified and strategies for retinal repair and regeneration by activation and differentiation of Müller stem cells into retinal neurons may be developed in the future. Recent studies have provided evidence that hMSC can be transplanted and successfully integrate into the neural retina of animal models of retinal degeneration [29,31,32,33]. Transplantation of hMSC-derived cells for the replacement of retinal neurons may therefore represent a realistic treatment target for the near future, with autologous transplantation also a possibility.

## 3. Replacement of Retinal Pigment Epithelium

### 3.1. Rationale for RPE Cell Replacement

RPE cells, which form a monolayer of pigmented cells adjacent to the photoreceptor outer segments, provide crucial support for the integrity and function of the adjoining retinal neurons by supplying nutrients such as glucose, omega-3 fatty acids and retinol, maintaining the homeostasis of the extracellular ionic environment as well as phagocytosing shed photoreceptor outer segments [34,35]. Abnormalities of the RPE have been shown to result in the progressive dysfunction and secondary degeneration of photoreceptors in a range of retinal degenerative diseases [36]. The pathophysiology is one of the leading causes of legal blindness in England and Wales [37], with age-related macular degeneration (AMD) attributable to abnormal RPE function [37].

Since RPE cells represent a uniform monolayer of cells which reside beneath the retina and do not involve any synaptic connectivity, their replacement may prove less complex than that of the interconnecting retinal neurons. However, in order to preserve retinal function, RPE cell transplantation must take place before the photoreceptors are irrevocably damaged and vision is permanently lost. Autologous subretinal translocation of RPE-choroid sheets or transplantation of RPE cell suspensions, which have been demonstrated to improve vision in patients with AMD, may serve as a proof of concept that the replacement of dysfunctional RPE can slow or prevent blindness due to RPE insufficiency [38]. In addition macular translocation surgery has been shown to restore visual function in patients with AMD [39]. RPE has been derived *in vitro* from iPS cells and hESC and the first clinical trials investigating the transplantation of hESC-derived RPE cells have recently commenced [40,41,42].

### 3.2. Derivation of Retinal Pigmented Epithelial Cells from Stem Cells

Studies have shown that transplantation of embryonic retinal sheets into the subretinal space can preserve photoreceptors in dystrophic rats and partly preserve retinal responses to light stimulation [43]. These findings suggest that grafting of healthy RPE cells may be able to support photoreceptors in a model of RPE insufficiency and indicate that the transplantation of hESC or iPS-derived cells may exert a similar effect on the neural retina in human RPE disease.

The generation of RPE from hESC has been extensively described. These cells display RPE-like morphology and express molecular markers typically expressed by RPE cells, such as RPE65, CRALBP, bestrophin, Mitf and PEDF [44,45,46,47,48,49,50,51]. In addition hESC-derived RPE cells have been shown to release the neuroprotective PEDF [48] and phagocytose photoreceptor segments [40], crucial functions of the RPE *in vivo*. hESC have been reported to differentiate into structures which resemble the optic cup during embryonic development and which contain RPE-like cells in their outer layer [52,53]. Following the subretinal transplantation of hESC-derived RPE cells into RCS dystrophic rats, a model of RPE insufficiency, the grafted cells were demonstrated to be retained [44,47] and retinal function to be improved in the transplanted eye [46,49]. 

Following subretinal transplantation human iPS cell-derived RPE cells have been shown to protect the retina in a rodent model of RPE insufficiency and secondary photoreceptor degeneration [41]. Human fibroblast-derived iPS cells have recently been differentiated towards cells with an RPE cell phenotype. These RPE-like cells are pigmented and display the typical regular polygonal morphology observed in cells of the adult RPE layer. They express a gene profile which is consistent with differentiation towards RPE cells and gain the ability to extensively phagocytose extracellular materials, including photoreceptor outer segments [41,54,55,56]. In addition a protocol has been established in the monkey for the generation of iPS cells from skin fibroblasts and subsequent differentiation towards RPE cells for autologous transplantation [57].

Recently, a self-renewing subpopulation of RPE cells has been isolated from the human eye. Although these cells can express markers of RPE cells as well mesenchymal and retinal neuronal cell types under differentiating cell culture conditions, no evidence, however, has been provided that they can give rise to functional RPE-like cells [58].

### 3.3. Clinical Trials using Stem Cell-Derived RPE

The first clinical trials investigating the transplantation of hESC-derived RPE have been recently approved by the European and American regulatory authorities and began recruitment in 2011. These phase 1A/B trials involve patients with either dry AMD [59] or Stargardt’s macular dystrophy [60] and are designed to test the safety and tolerability of grafted hESC-derived RPE cells. Although primarily designed as safety studies, functional outcome measures such as best corrected visual acuity, multifocal ERG, reading speed as well as structural evidence from optical coherence tomography (OCT) and autofluorescence imaging are being recorded. More recently, a further clinical safety study has been started for the transplantation of hESC-derived RPE cells into patients with Stargardt’s macular dystrophy, fundus flavimaculatus and juvenile macular dystrophy [61]. 

The first data from two of these clinical trials has been reported 4 months after transplantation, with cells reported to persist without signs of rejection and to be safe in the absence of evident hyperproliferation or tumorigenesis. Although functional recovery has been reported in both patients receiving hESC-derived RPE to date, this finding may be confounded by an observed improvement in the non-transplanted fellow eye [42]. Nevertheless, the reporting interval of these clinical trials was short and any functional improvement may be more pronounced when the studies are scheduled to end one year after transplantation in July 2013.

## 4. Repairing the Neural Retina: Photoreceptors

### 4.1. The Worldwide Burden of Photoreceptor Degeneration

Retinal disease, which ultimately leads to the degeneration of photoreceptors, is the third leading cause of worldwide blindness [1]. The degeneration of photoreceptors observed in AMD and diabetic retinopathy is secondary in nature, with abnormalities in RPE function being the significant primary disorder [62,63]. In these conditions, replacement of photoreceptors with stem cell-derived precursors is only likely to be successful once healthy RPE has been restored. Mutations within the rhodopsin gene account for over thirty percent of cases of autosomal dominant RP, with more than 80 different mutations having been identified [64]. Progressive and permanent visual loss is also a cardinal feature of such inherited primary photoreceptor degenerations, which are reported to affect approximately 1 in 3,000 people [65]. The development of strategies for photoreceptor replacement provide an exciting prospect for the restoration of sight for individuals in this group whose vision has been significantly damaged and for whom no treatments are currently available. 

Repairing the neural retina may be regarded as a more complex task than that of replacing the RPE, since it requires re-establishing of synaptic connections. In the early stages of retinal degeneration, replacement of abnormal photoreceptors may be sufficient to maintain retinal function if transplanted cells are able to form functional synaptic connections with host bipolar cells. Synaptic remodeling of neural circuits is known to occur, however, as retinal degeneration progresses [66], resulting in a complex non-functional neural network into which the integration of transplanted cells may prove difficult. Nevertheless the observation of residual plasticity earlier in the disease course [66] may indicate a window of opportunity for neuronal replacement strategies. In addition the replacement of a unidirectional sensory neuron, such as the photoreceptor, can be expected to be more straightforward than that of second and third order retinal neurons such as RGCs, which receive numerous afferent inputs and distant synapses within the brain [67]. 

### 4.2. Derivation of Photoreceptors from Embryonic Stem Cells

Early work involved the transplantation of undifferentiated ESCs into the subretinal space of animal models. It was observed that ESCs appeared to show enhanced migration and integration in eyes with damaged retina when compared to transplantation beneath healthy retina [68]. It was also hypothesized that the microenvironment in the subretinal space may promote the differentiation of transplanted cells towards mature photoreceptors [69] and that intrinsic signaling processes may play a role in cell fate determination [70]. 

Contemporary research has challenged the concept that undifferentiated stem cells are the ideal candidates for transplantation strategies aimed at neuronal replacement. A landmark study defined the optimal developmental stage for successful rod photoreceptor transplantation in the mouse, demonstrating both structural integration and evidence of functional improvement using pupillometry following subretinal transplantation [71]. A recent report describes synaptic connectivity with higher cortical centers and improved visual function as seen by optokinetic head tracking, following subretinal transplantation of similar photoreceptor precursors in the mouse [72].

The ontogenetic stage of committed rod photoreceptor precursors suitable for transplantation was suggested to be at the stage of NRL (Neural Retina Leucine Zipper) expression. This finding will prove difficult to translate into human therapy, however, as equivalent donor cells would need to be obtained from a human foetus in the second trimester. Although recent reports have supported the observation that newly formed rods are able to successfully integrate within the host retina, they have conversely found that although fully mature rods exhibit a higher rate of transplant failure, the ontogenetic stage of the donor cell appears to be less critical than previously suggested [73].

In order to facilitate the development of human therapies, the lessons learnt from rodent studies have begun to be applied to the development of protocols for the differentiation of embryonic stem cells of human origin *in vitro*, in order to provide a potential source of cells for transplantation. hESCs have been shown to differentiate *in vitro* into retinal progenitor cells which primarily formed inner retinal neurons, but upon co-culture with mouse retina were shown to increase the expression of photoreceptor markers [7]. Subsequently protocols were published demonstrating the stepwise differentiation of hESCs into retinal progenitors, followed by treatment with retinoic acid and taurine which induced the expression of photoreceptor markers *in vitro* after 150 days [11,74]. Optimization of a stepwise photoreceptor differentiation protocol has led to the significant shortening of the time required for the generation of photoreceptor progenitors from hESC [75]. Recently, the derivation of cells with functional photoreceptor characteristics from hESC has been reported [50].

The potential exhibited by *in vitro* work is underlined further by transplantation studies with hESC-derived precursors in rodent models of photoreceptor damage. When delivered into the subretinal space of a mouse model of Leber’s Congenital Amaurosis, transplanted cells integrated into the host outer nuclear layer with partial restoration of the b-wave of the electroretinogram [76]. Similarly, the transplantation of hESC-derived photoreceptor progenitors into a rabbit model of RPE damage resulted in a slight improvement of the scotopic and photopic b-waves [77].

### 4.3. Derivation of Photoreceptors from iPS Cells

The generation of photoreceptor precursors from iPS cells which exhibit biological behavior indistinguishable from that of ESCs [15] has become the focus of much recent work, with the ultimate aim of producing patient-specific cells for transplantation.

*In vitro* suspension culture of human iPS cells with Wnt and Nodal antagonists has been shown to induce the expression of markers of retinal progenitor cells and generate RPE cells. Subsequent treatment with retinoic acid and taurine was able to upregulate the expression of markers of photoreceptors [78]. Alternative human iPS cell differentiation protocols employing bone morphogenetic protein (BMP)-4 and Wnt antagonists in conjunction with insulin-like growth factor 1 (IGF-1) treatment have been shown to facilitate the induction of photoreceptor markers *in vitro* [10]. An enriched population of photoreceptor precursors, which was selected using GFP expression driven by IRBP cis-regulatory sequences, was found to integrate into the outer nuclear layer of wild type mice and express markers of photoreceptors *in vivo*. 

Both hESCs and human iPS cells have demonstrated considerable potential to be taken forward to clinical trials of photoreceptor diseases, but are currently limited by concerns regarding their long term safety [12,19]. Whilst work continues in order to address these concerns, complementary research into alternative sources of photoreceptors for transplantation may circumvent these issues.

### 4.4. Adult Müller Stem Cell-Derived Photoreceptors

The concept that Müller stem cells from within the adult mammalian retina may generate new neurons *in vivo* has been introduced earlier and is thoroughly reviewed by Jadhav *et al.* [79]. However, it is worthwhile evaluating the evidence, which supports the hypothesis that such cells may play a role in the generation of photoreceptors.

Evidence of endogenous activation of Müller stem cells in the adult mammalian retina was demonstrated following experiments using N-methyl-N-nitrosourea (MNU) to deplete host photoreceptors [80]. In response to this specific injury, Müller glia were observed to proliferate with a concomitant increase in Nestin expression, a marker of neural stem cells. In addition, Müller glia-derived cells expressing rhodopsin were observed in the outer nuclear layer. These observations suggest that not only could Müller glia be a potential source of new retinal neurons by endogenous reactivation, but that they may have role as a transplantation candidate for photoreceptor replacement strategies. The findings would suggest that the neurogenic property of Müller cells to facilitate photoreceptor regeneration in lower vertebrates [23] may be conserved in the adult rodent retina both *in vitro* and *in vivo*.

Similar data examining Müller stem cells from the adult human retina *in vitro* are however relatively sparse in the literature. A recent study provided the first evidence that Müller stem cells isolated from the adult human retina may be differentiated *in vitro* towards a photoreceptor fate [81]. Transplantation of these cells into the rodent retina showed limited integration into the outer nuclear layer without evidence of synaptic connectivity. Further work in our laboratory has demonstrated that hMSC-derived photoreceptor precursors are able to partially restore the a-wave of the electroretinogram following transplantation into a rodent model of primary photoreceptor degeneration [82].

## 5. Repairing the Neural Retina: Retinal Ganglion Cells

### 5.1. Diseases Affecting Retinal Ganglion Cells

Glaucoma, a progressive optic neuropathy usually associated with elevated intraocular pressure and characteristic visual field defects, is characterized by the loss of retinal ganglion cells (RGCs). Raised intraocular pressure is thought to lead to the damage of RGC axons as they exit the eye at the optic nerve head. If left untreated, this results in loss of RGCs which comprise the optic nerve and ultimately leads to blindness [83]. Although the exact mechanisms of RGC damage and loss are not fully understood, a range of factors have been suggested to contribute to RGC damage, including the impairment of the retrograde axonal transport of neurotrophic factors as well as involvement of astrocytes, which lead to apoptotic RGC death [84,85]. In addition, evidence has been presented that reduced nerve fiber layer thickness is observed in other conditions such as Alzheimer’s disease [86] or in diabetes with minimal proliferative diabetic retinopathy [87]. However glaucomatous retinal damage is restricted predominantly to RGCs [83], and patients may still maintain functional vision and good quality of life even with only a relatively small number of functioning RGC axons. Therefore providing support to the surviving RGCs as well as their replacement in end-stage glaucoma would appear to be an effective target for stem cell-based treatment strategies.

### 5.2. Retinal Ganglion Cell Replacement Strategies with hESC

Various studies have provided evidence that embryonic stem cells (ESC) can differentiate into cells with retinal neural characteristics, including RGC-like cells. By exposing murine ESC to various growth and differentiation factors, they can be induced to express typical RGC markers such as *Ath5*, *Brn3b*, *RPF-1*, *Thy-1* and *Isl-1* [88]. Rx/rax-expressing murine ESC, which have been differentiated towards neural cells by treatment with retinoic acid, display molecular markers of RGCs and horizontal cells. In addition these cells acquire an electrophysiological phenotype, which is consistent with RGCs, and integrate *ex vivo* into the mouse retina [89]. Similarly, Aoki *et al.* demonstrated that murine ESC may be differentiated into eye-like structures, which express RGC markers like BRN3B and TUJ1 upon grafting into the host eye. However, electrophysiological analysis of the transplanted eyes revealed that the grafted cells did not improve optic nerve function. In addition, the transplanted cells were shown to hyperproliferate and form teratoma-like structures [90].

Although most studies investigating the potential of ESC for the generation of RGCs have employed cells of animal origin, hESC have also been reported to be an appropriate source for inner retinal neurons. Using a suitable differentiation protocol hESC have been shown to differentiate into retinal precursors, as indicated by the expression of PAX6 and CHX10. Further differentiation yielded a high percentage of inner retinal neurons, including cells with characteristics of RGCs [7]. It has been shown that neuralized hESC can integrate into the retina and adopt the morphology and markers of various retinal neurons following intravitreal transplantation in mice, including those of inner retinal neurons [91]. Although some studies have mainly reported differentiation of hESC into cells expressing photoreceptor markers, these cells were nevertheless shown to survive in the mouse retina for extended periods after subretinal and intravitreal transplantation [69]. Interestingly, hESC differentiated towards retinal progenitor cells were able to integrate into the RGC layer of the retina following epiretinal transplantation and survived without immunosuppression [92]. 

### 5.3. iPS Cell-Derived Retinal Ganglion Cells

The transplantation of iPS cell-derived RGCs has recently been investigated, since they may provide the possibility for autologous transplantation while at the same time sharing many characteristics of hESC. iPS cells derived from murine fibroblast by the transfection of *Oct3/4*, *Klf4*, *Sox2* and *c-Myc* have been shown to form retinal neurons including RGC-like cells. Neural induction and exposure to conditioned media from E14 rat retinal cells were required to augment expression of important regulators of RGC development, such as *Ath1*, *Brn3b* and *Rpf1* and *Irx2*, which simultaneously led to the downregulation of markers of retinal progenitors, including *Sox2* and *Chx10*. The resulting iPS cell-derived RGCs were shown to express functional voltage-gated sodium channels, which are characteristic of neural cells including RGC [93].

Similarly, mouse iPS cells from reprogrammed mouse fibroblasts could be differentiated by attenuation of the Wnt, Notch1 and BMP pathways towards RGC-like cells, as demonstrated by the upregulation of specific genes such as *Brn3b*, *Islet-1* and *Thy1.2*. However, these iPS cell-derived RGCs only rarely integrated into the retina following intravitreal transplantation and may not have lost their ability to self-renew, resulting in the formation of teratomas [94].

As described above, major issues associated with iPS cells remain to be resolved before they can be used in clinical applications. Above all their safety profile remains to be investigated due to the risk of tumorigenesis, while the generation and validation of patient-specific iPS cells for autologous transplantation may prove to be costly and time consuming. In addition, the successful transplantation of functional iPS cell-derived RGCs remains to be demonstrated.

### 5.4. Retinal Ganglion Cell Replacement Using Human Müller Stem Cells

hMSC, which have been isolated and cultured from human cadaveric retina [29], can proliferate indefinitely and express various markers of retinal progenitors such as Notch1, *SOX2*, *PAX6*, *SHH* and *CHX10*. In addition they display proteins typical of Müller glia as well as of retinal neurons like vimentin, CRALBP, HUD, PKC, peripherin and opsin [29], indicating that they are committed to a retinal neuronal phenotype. Differentiation of hMSC by Notch1 inhibition has been shown to lead to the enrichment of a cell population with a phenotype resembling that of RGC precursors, as indicated by a change in morphology towards a neural appearance as well as the upregulation of genes and proteins such as BRN3b, ISL1 and βIII-tubulin [33].

The majority of studies investigating the transplantation of hMSC into the retina have employed undifferentiated cells. Nevertheless successful engraftment and integration of hMSC following subretinal injection has been demonstrated in neonatal Lister Hooded rats or adult dystrophic RCS rats [29]. In addition transplanted cells expressed markers of retinal neurons such as the RGC marker HUD as well as calretinin, which is characteristic for RGCs and amacrine cells [29]. It has been observed, however, that undifferentiated hMSC migrate into all layers of the retina and show no specificity for RGC morphology [31]. Interestingly, following the transplantation into a rodent model of glaucoma, only a minor proportion of hMSC expressed the Müller glia and astrocyte marker GFAP, while several cells expressed the neuronal protein βIII-tubulin. It is important to note that a significant proportion of the transplanted cells appeared to migrate towards the host retina [32].

Most recently hMSC-derived RGC precursors have been successfully transplanted into a rat model of RGC depletion. These cells were shown to upregulate markers of RGC, such as ISL-1, BRN3B and HUD, and to express functional nicotinic acetylcholine receptors, a hallmark of neuronal cells including RGC. Importantly, transplantation of these RGC-committed hMSC led to the selective integration of the grafted cells into the nerve fiber layer of the host. Significantly, partial restoration of of RGC function was also demonstrated by scotopic electroretinography in transplanted animals [33].

Crucially these most recent findings provide proof of concept that transplantation of hMSC-derived RGC precursors are able to integrate into the retina after RGC damage and subsequently ameliorate the effects of RGC damage on retinal function in a small animal model. Further studies in larger eyes will likely be needed in order to optimize cell delivery methods onto the inner retinal surface. In addition safety studies must be successfully concluded before clinical trials may begin. Since the autologous derivation of hMSC from a diseased environment may be unlikely to succeed and would prove to be time-consuming and costly, the setup of tissue banks for allogeneic transplantation may be a suitable alternative. In summary, the transplantation of hMSC-derived RGC precursors is currently the most advanced approach for functional RGC replacement and merits first in man exploratory studies. 

### 5.5. Optic Nerve Regeneration

Regeneration of the optic nerve requires extension of new RGC axons through the lamina cribrosa and existing myelinated nerve to synapse appropriately in the multilayered lateral geniculate nucleus. This approach would not only require an appropriate source of RGC precursors but also methods to induce axon growth and guidance as well as the formation of appropriate synapses to restore the functional circuitry. There has been considerable work over the past two decades into promoting the survival and regeneration of existing mature RGC axons following injury [95], and lessons learnt from this approach may help facilitate optic nerve regeneration from stem cells in the future.

Work in axotomy models has shown that RGC neurite outgrowth is attenuated by glycoproteins associated with the Schwann cell complex [96], although this inhibition can be overcome by a synergistic combination of neurotrophic factors [97]. While significant regeneration of the optic nerve is yet to be seen in rodent models, neural progenitor cells have been observed to migrate through the lamina cribrosa and demonstrate axon growth into the optic nerve [98,99]. Neurotrophic factors have also been shown to enhance RGC axon growth *in vivo*. Following axotomy, expression of fibroblast growth factor 2 via gene therapy techniques led to increased axon elongation in the mouse [100]. A similar observation was reported in response to administration of ciliary neurotrophic factor (CNTF), with the promotion of axon outgrowth *in vivo* and stimulation of resident astrocytes to produce further CNTF [101]. Distinct proteins secreted by activated macrophages have also been associated with RGC axon elongation [102] and inhibition of axonal outgrowth in the spinal cord has been observed following administration of retinoic acid receptor agonists [103]. 

The glial scar that forms following injury to the CNS [104] provides an additional physical barrier which needs to be overcome. These areas of scarring are rich in chondroitin sulphate proteoglycans (CSPGs) [105], that behave in an inhibitory manner. Within the CNS, CSPGs are known to inhibit axon guidance following injury [106] and in the rat eye they have been reported to inhibit the regeneration of the optic nerve following injury [107]. CSPGs are thought to play a role in axon guidance during normal retinal development and have been demonstrated to inhibit neurite outgrowth from rat RGC *in vitro* [108]. The enzymatic degradation of CSPGs within the glial scar using chondroitinase has been shown to enhance neurite outgrowth following injury both within the brain [109] and spinal cord [110,111]. Similarly digestion of CSPGs using chondroitinase ABC (ChABC) was reported to facilitate the migration of Müller stem cells across the retina following transplantation [31] and therefore matrix modulation in this manner is likely to facilitate the migration of RGC axons towards their distal first synapse in the context of optic nerve regeneration.

The rho-associated kinase (ROCK) pathway is integral to the mechanisms of axon regeneration, neurite outgrowth and the projection of neurons within the CNS [112,113,114]. Other molecules previously implicated with the inhibition of axon regeneration, such as Nogo-A, myelin-associated glycoprotein (MAG) and oligodendrocyte-myelin glycoprotein (OMGP) have been shown to exert their inhibition of axon growth by activation of the Rho-mediated signal transduction pathway [115,116,117]. Pharmacological ROCK inhibition led to increased neurite outgrowth *in vitro* and a dose-dependent increase of RGC axonal regeneration was also observed in a rodent optic nerve crush model [118]. This effect of ROCK inhibition was synergistically enhanced by administration of CNTF, a potent neurotrophic factor for retinal ganglion cells, with enhanced RGC survival observed in both *in vitro* and *in vivo* models [119]. Further studies into the ROCK pathway may enhance current knowledge of the inhibitory signals that prevent axonal regeneration *in vivo* and enhance future models of *in vivo* optic nerve regeneration.

The mechanisms and pathways involved in optic nerve regeneration are clearly complex and results to date have been limited. However induction of long distance axon regeneration in the mouse has been observed in a complex experimental environment involving an inflammatory response, elevated cAMP and a gene deletion [120]. These reports described half of the axons crossing the optic chiasm with a small subset reaching the thalamus. Further work from the same group earlier this year observed the extension of regenerating RGC axons to several midbrain structures [121], making this first report of RGC regeneration leading to synaptic connectivity with the brain. These findings were accompanied by functional improvement using RGC mediated pathways, including improved optomotor and pupillomotor responses, depth perception and photoentrainment of circadian activity in the treated animals. 

There is still a burden of work required in order to translate these findings to the anatomically longer distances that occur in the human visual system. However it the current literature suggests that regeneration of the optic nerve is a step closer and with several potential pathways identified that require further study in order to make this a reality.

## 6. Assessment of Retinal Function and Structure Following Stem Cell Transplantation

The recent advances in the development of cell replacement strategies for retinal degenerative conditions necessitates the use of techniques which can evaluate retinal structure and function in order to assess the outcomes of stem cell transplantation. A range of methods for the structural analysis of the retina *in vivo* as well as retinal and optic nerve function are already widely used to examine retinal damage in the clinic. These techniques are often adaptable to experimental settings and therefore may be used both for clinical purposes as well as for preclinical and clinical trials. Retinal structure is usually measured by optical coherence tomography (OCT), while functional outcome measures include the Ganzfeld, multifocal and pattern electroretinogram (ERG), visual evoked potential (VEP) and pupillometry.

The ERG, which measures electrical impulses and sum potentials generated by various retinal cells, is currently widely used in the laboratory and in clinic to determine retinal function and will likely play a key role in the evaluation of novel therapeutic approaches using stem cell transplantation for the treatment of retinal degenerative conditions. Although ISCEV guidelines have been published, which standardize ERG protocols in the clinic [122,123,124], these can be readily adapted and complemented for specialized experimental purposes. The scotopic and photopic Ganzfeld flash ERGs are generally used to evaluate rod and cone photoreceptor function, respectively [122], while the multifocal ERG quantifies the ERG response evoked in specific areas of the retina [124]. Conversely, the pattern ERG, which often employs an alternating black and white checkerboard stimulus, is more widely used to assess RGC damage and optic nerve dysfunction, as observed in glaucoma and other optic neuropathies [125,126]. By applying different check sizes, the pattern ERG can also estimate the extent of glaucomatous damages [127].

While the Ganzfeld and multifocal ERGs are extensively used in both preclinical and clinical trials due to their relative ease of use, the pattern ERG is rarely applied in animal studies and mostly restricted to clinical settings, since it requires adequate accommodation and fixation [123]. The multifocal pattern ERG has recently been described and shown to be attenuated in glaucoma patients with reduced RGC numbers [128,129]. Whether it gains widespread use in the assessment of RGC transplantation, however, remains to be seen, since localized attenuation of the signal amplitude was not associated with visual field losses [130]. In order to assess RGC function in an experimental setting the scotopic threshold and photopic negative responses are frequently used, although they may contain contributions from other retinal neurons, such as glia and amacrine cells [131,132,133].

In the clinic, RGC function and the integrity of the optic nerve are usually evaluated by pattern reversal, pattern onset/offset or flash VEP [134]. Of these techniques the flash as well as pattern onset/offset VEP have proved to be useful for preclinical animal studies [135,136]. Although multifocal mapping of VEP has recently been introduced [137,138], this technique is currently not routinely used either clinically or the laboratory.

## 7. Conclusions

Development of stem cell-based therapies has been the focus of intense research and may benefit large numbers of patients with visual impairments due to retinal disease in the years ahead. Great advances have recently been made towards the generation of stem cell transplantation techniques for the functional replacement of RPE cells, photoreceptors and RGCs. The development of RPE cells from hESC is currently at the most advanced stage with clinical safety studies having commenced within the last year. In addition, the development of cell replacement strategies for both photoreceptors and RGC using precursor cells derived from the less controversial hMSC has recently been reported in animal studies, with the possibility of exploring these cells further for the development of clinical trials in the near future. Since the autologous transplantation of individual patient-derived iPS cells and adult stem cells will likely be costly, their clinical application may require the creation of tissue banks for donor cells. These recent exciting developments in the functional replacement of retinal neurons and RPE cells, give rise to the expectation that clinical replacement of damaged retinal cells may be able to alleviate the consequences of retinal degenerative disease in the near future.
